# MIL-101 (Fe) @Ag Rapid Synergistic Antimicrobial and Biosafety Evaluation of Nanomaterials

**DOI:** 10.3390/molecules27113497

**Published:** 2022-05-29

**Authors:** Xi Li, Huiying Zheng, Jiehan Chen, Mengyuan Xu, Yan Bai, Tiantian Liu

**Affiliations:** School of Public Health, Guangdong Pharmaceutical University, Guangzhou 510310, China; lixi8813@163.com (X.L.); zhyzhy_2021@163.com (H.Z.); chenjie.han@163.com (J.C.); 18762176339@163.com (M.X.); angell_bai@163.com (Y.B.)

**Keywords:** metal-organic framework, Ag nanoparticles, hybrid nanoagents, antibacterial activity

## Abstract

Metal-organic frameworks (MOFs), which have become popular in recent years as excellent carriers of drugs and biomimetic materials, have provided new research ideas for fighting pathogenic bacterial infections. Although various antimicrobial metal ions can be added to MOFs with physical methods, such as impregnation, to inhibit bacterial multiplication, this is inefficient and has many problems, such as an uneven distribution of antimicrobial ions in the MOF and the need for the simultaneous addition of large doses of metal ions. Here, we report on the use of MIL-101(Fe)@Ag with efficient metal-ion release and strong antimicrobial efficiency for co-sterilization. Fe-based MIL-101(Fe) was synthesized, and then Ag^+^ was uniformly introduced into the MOF by the substitution of Ag^+^ for Fe^3+^. Scanning electron microscopy, powder X-ray diffraction (PXRD) Fourier transform infrared spectroscopy, and thermogravimetric analysis were used to investigate the synthesized MIL-101(Fe)@Ag. The characteristic peaks of MIL-101(Fe) and silver ions could be clearly seen in the PXRD pattern. Comparing the diffraction peaks of the simulated PXRD patterns clearly showed that MIL-101(Fe) was successfully constructed and silver ions were successfully loaded into MIL-101(Fe) to synthesize an MOF with a bimetallic structure, that is, the target product MIL-101(Fe)@Ag. The antibacterial mechanism of the MOF material was also investigated. MIL-101(Fe)@Ag exhibited low cytotoxicity, so it has potential applications in the biological field. Overall, MIL-101(Fe)@Ag is an easily fabricated structurally engineered nanocomposite with broad-spectrum bactericidal activity.

## 1. Introduction

Pathogenic bacterial infections have become one of the most serious problems threatening public health. Many diseases caused by pathogens, such as intestinal infections and lung inflammation, affect the lives and health of people worldwide. Since the end of 2019, the outbreak of COVID-19 has posed a great risk to human health and economies worldwide, with co-infections of viruses and bacteria occurring in several countries against the backdrop of continuous viral mutations [1,2,3,4]. Recent studies have shown that infections caused by *Escherichia coli* and *Staphylococcus aureus* are important factors in human pathogenesis [5,6]. *E. coli* is a Gram-negative bacterium with a short bacillus with blunt rounded ends. It is a conditional pathogen, and under certain conditions, *E. coli* infections can occur in the gastrointestinal tract, urinary tract, and other local tissues and organs [7]. *S. aureus* belongs to the genus Staphylococcus, and it is a Gram-positive bacterium. *S. aureus* pneumonia is serious, and it is one of the main causes of purulent lung infections [8]. In the last few decades, various antibiotics have been developed to prevent and fight infections. There are various types of anti-infective drugs, including β-lactam antibiotics, aminoglycoside antibiotics, macrolide antibiotics and quinolones, and antifungal drugs such as clotrimazole and ketoconazole [4,5]. However, these anti-infective drugs have drawbacks, and they can cause are many adverse reactions, including drug fever, rashes, intestinal reactions, anaphylaxis, contact dermatitis, photosensitive reactions, secondary infections, and angioneurotic edema. Moreover, the treatment of pathogenic bacterial infections with antibiotics has become very difficult due to the chronic misuse of antibiotics, which has increased bacterial resistance and is now a clinical problem. Therefore, it is important to develop innovative antimicrobial systems to treat bacterial infections [6,7,8].

In recent years, instead of antibiotics, new materials such as inorganic nanocomposites and antibacterial peptides have gradually begun to be reported as antibacterial materials in the biological field. However, these materials have their own drawbacks. For example, their single antibacterial mechanism can only maintain a limited bactericidal rate, and they also have a certain toxicity. Therefore, it is urgent to design a dual-mechanism antibacterial material to fight pathogenic bacteria. Metal–organic frameworks (MOFs) are formed by chemically aligning organic ligands to central metal ions to construct porous three-dimensional framework structures [9,10,11,12,13,14,15,16,17,18]. In current research, MOF materials are mainly used in energy-storage, gas adsorption and separation, catalysis, sensing, magnetism, and fluorescence applications. Carrillo-Carrión [19] reported that MOFs have emerged as one of the most fascinating libraries of porous materials with a large potential in very diverse application areas. Huang et al. [20] synthesized bimetallic Ce–Ni MOFs (Ce–Ni-MOFs) via hydrothermal reactions using 1,3,5-benzenetricarboxylic acid as a ligand. Yi et al. discovered that MIL-53(Fe) MOFs can significantly enhance the chemiluminescence of luminol in the presence of H_2_O_2_ in alkaline media. This finding led to a new chemiluminescence method for the biosensing of glucose when combined with glucose oxidase, confirming the existing application of MOF materials [21].

With the increasing research on nanotechnology, nanomaterials are considered to be the most promising antimicrobial agents. They not only play an important role in photothermal and antimicrobial therapies but also show good biosafety properties. Gold, silver, zinc, and their compounds are common antimicrobial agents. Among these elements, Gold gold and nanosilver are the most common antimicrobial agents, and they are more widely used due to their long-lasting bactericidal effects [14,15]. Silver nitrate was used as an antibacterial material for the treatment of venereal diseases and other disorders in the 18th century. The silver ions contained in silver nitrate are an excellent antimicrobial agent, but the use of silver nitrate is limited due to its inherent toxicity. With increasing use in various fields such as clinical nanotechnology and medicine, Sobhan Mortazavi-Derazkola et al. synthesized CME@Ag-NPs in a green way from a leaflet fruit extract, and they biosynthesized CME@Ag-NPs against multidrug-resistant human pathogens [16]. The bacteria exhibited excellent antibacterial efficiency and showed significant anticancer activity in AGS and MCF-7 cell lines. Hakimeh Teymourinia et al. used cotton–silver–graphene quantum dots (cotton/Ag/GQDs) nanocomposites as novel antibacterial nanomats [17]. Razieh Razavi et al. utilized the bio-oil-in-water nanoemulsion technique to freely synthesize silver nanoparticles (AgNPs), which are simple, green, economical, and have low toxicity to cells [18]. A low cytotoxicity or non-toxic antimicrobial material needs to be established and then be developed to improve its take advantage of and transformed into a prevention and control product.

MOFs have high porosity and large specific surface area, and the open metal sites have certain antibacterial capabilities [21,22,23,24]. In addition, due to their good biocompatibility, MOFs are candidates for loading drugs and metal particles [25,26,27]. Combining MOF materials with other organic and inorganic materials has become a current research trend. Hu et al. reported on an organic framework containing silver metal that showed excellent antibacterial activity [28]. Huang et al. successfully loaded Ag ions as a typical antibacterial agent into MIL-53(Fe) through simple impregnation, forming MIL-53(Fe)@Ag with antibacterial activity and low cytotoxicity [29]. The free silver ions in MIL-53(Fe)@Ag can directly inactivate the basic proteins in the bacterial cell and then kill bacteria. Therefore, MOFs are suitable for producing antibacterial nanosystems. Although adding metal ions can enhance the sterilization ability of MOFs, this process also has some shortcomings, such as the cytotoxicity caused by high doses of metal ions, slow sterilization speed, and an unsatisfactory sterilization effect [30,31,32,33,34,35,36].

In this study, we designed a derivative of the structurally engineered Ag^+^-doped MOF MIL-101(Fe)@Ag that has a high-efficiency ion-release capacity, antibacterial properties, reliable biosafety, and can be used for broad-spectrum bacterial sterilization. We prepared a novel type of functionalized MIL-101(Fe)@Ag nanocomposite; characterized the structure of the material with powder X-ray diffraction (XRD), scanning electron microscopy (SEM), and Fourier transform infrared (FTIR) spectroscopy; and investigated the mechanism of MIL-101(Fe)@Ag synthesis from the characterization results. The antibacterial properties of MIL-101(Fe)@Ag against *E. coli* and *S. aureus* were investigated with bacterial growth curves and plate-coating experiments. The reactive oxygen species (ROS) assay was used to explore the antibacterial mechanism, analyze the mechanism of action of the nanomaterials against bacteria, and explore the biosafety of the nanomaterials at the cellular level to lay the foundation for the development of new, safe, and effective antibacterial agents [37,38,39].

## 2. Results and Discussion

### 2.1. Synthesis and Characterization of MIL-101(Fe) and MIL-101(Fe)@Ag

**Figure 1:**
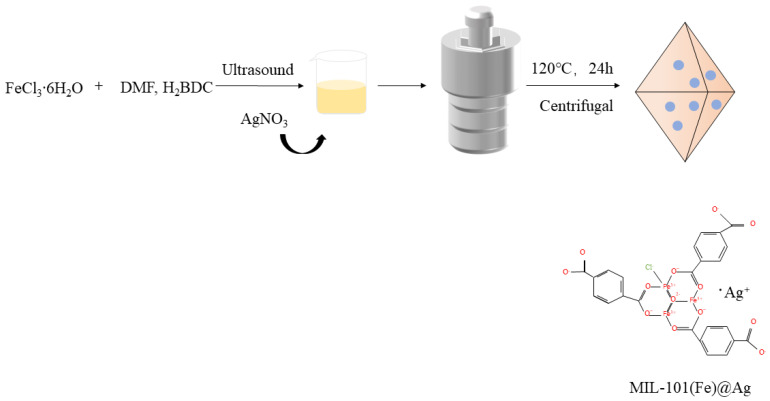
Experimental flow chart for synthesis of MIL-101(Fe)@Ag.

MIL-101(Fe)@Ag was synthesized with the solvothermal synthesis method. First, H_2_BDC and FeCl_3_∙6H_2_O were reacted in DMF, and then silver nitrate was added. The nanocomposites were obtained under high-temperature and high-pressure conditions in the reactor. A schematic diagram of the whole reaction process is shown in Figure 1. The laboratory-synthesized MIL-101(Fe)@Ag nanoparticles were investigated with powder XRD (PXRD), FTIR spectroscopy, ICP mass spectrometry, TG analysis, XPS, and the BET method [40].

#### 2.1.1. PXRD

**Figure 2:**
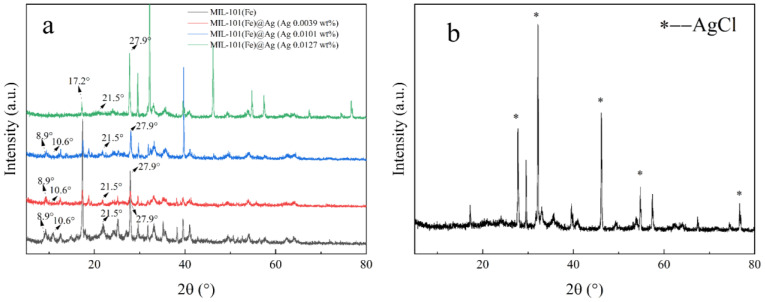
(**a**) Comparison of the PXRD spectra of MIL-101(Fe), MIL-101(Fe)@Ag (Ag 0.0039 wt%), MIL-101(Fe)@Ag (Ag 0.0101 wt%), and MIL-101(Fe)@Ag (Ag 0.0127 wt%). (**b**) PXRD spectrum of MIL-101(Fe)@Ag (Ag 0.0127 wt%).

PXRD can be used to accurately and efficiently detect the crystallinity of samples, and the structural characteristics of the samples can be determined by comparing the PXRD patterns of products (Figure 2) [41,42,43]. Here, the synthesized products were investigated with PXRD by varying the addition ratio of FeCl_3_∙6H_2_O and silver nitrate. The diffraction peaks (2θ) at 8.6°, 8.9°, 10.2°, 10.6°, 16.4°, 19.5°, and 21.5° in the PXRD pattern are the characteristic peaks of MIL-101(Fe) [44]. Comparing the diffraction peaks of the simulated PXRD patterns (Figure 2a) clear showed that MIL-101(Fe) was successfully constructed and silver ions were successfully loaded in MIL-101(Fe) to synthesize an MOF with a bimetallic structure, that is, the target product MIL-101(Fe)@Ag.

#### 2.1.2. FTIR Spectroscopy

**Figure 3:**
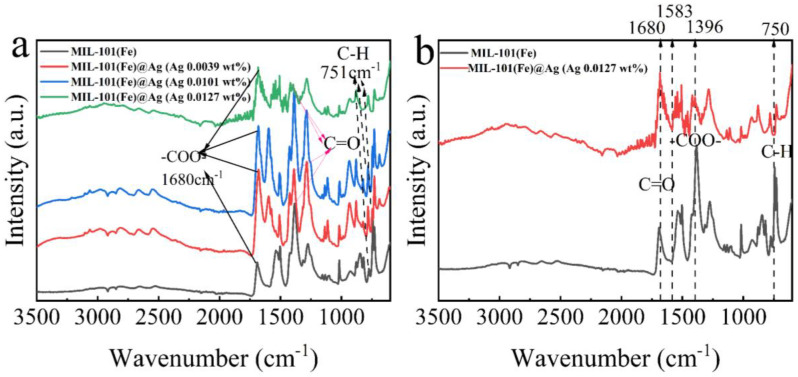
(**a**) FTIR spectra of MIL-101(Fe) and MIL-101(Fe)@Ag. (**b**) Comparison of the FTIR spectra of MIL-101(Fe) and MIL-101(Fe)@Ag (Ag 0.0127 wt%).

FTIR spectroscopy can be used to identify the functional groups of a sample by the positions of the functional-group types, and comparisons with MIL-101(Fe) could show whether the synthesized product was the desired product [45]. Here, the intensities of the peaks gradually decreased with increasing amount of AgNO_3_ (Figure 3b). This may have been because of the addition of AgNO_3_ during the synthesis of MIL-101(Fe)@Ag, which affected the synthesis of the product. Owing to the interaction force between Fe^3+^ and Ag^+^, together with the aggregation of Ag^+^, after the addition of more than 0.0127 wt% silver nitrate, a large number of silver ions aggregated and subsequently comprised the structure of the synthesized MOF collapse, and the characteristic peaks of the PXRD structure shifted. In the FTIR spectra of MIL-101(Fe)@Ag (Figure 3), the C–H bond of the benzene ring remained at 751 cm^−1^. The characteristic peaks at 1396 and 1583 cm^−1^ are the symmetric and asymmetric vibrations of the carboxyl group (–COO–), while the characteristic peak at 1680 cm^−1^ is associated with the presence of a C=O bond in the free carboxyl group, indicating the presence of a continuous dicarboxyl linkage [41,42,43,44]. This showed that the peaks of MIL-101(Fe) were still present in the FTIR spectrum of MIL-101(Fe) loaded with silver ions.

#### 2.1.3. ICP Analysis

**Figure 4:**
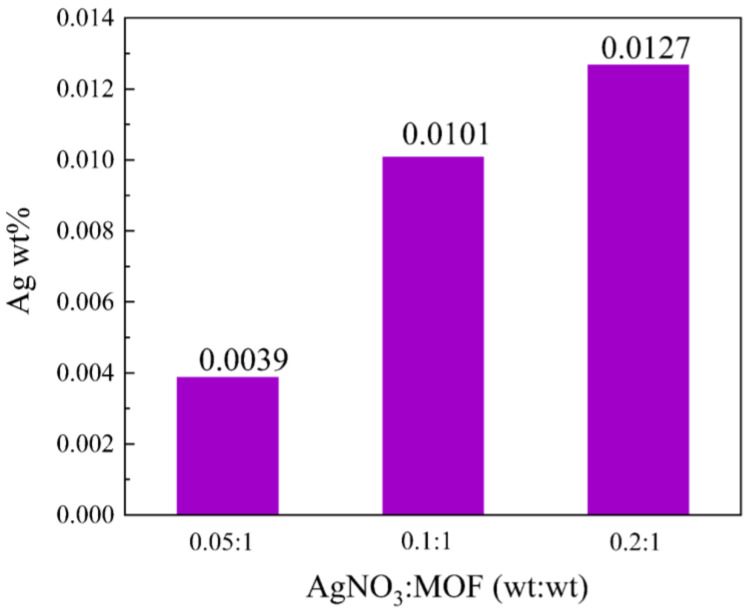
Ag-ion loading of MIL-101(Fe)@Ag with different mass ratios.

The loading capacity was then investigated with ICP mass spectrometry [46,47]. Products were synthesized with the addition of silver nitrate to MIL-101(Fe)@Ag of 0.0039 wt% (MIL-101(Fe)@Ag (Ag 0.0039 wt%)), 0.0101 wt% (MIL-101(Fe)@Ag (Ag 0.0101 wt%)), and 0.0127 wt% (MIL-101(Fe)@Ag (Ag 0.0127 wt%)). The maximum Ag-ion loading content of MIL-101(Fe)@Ag was 0.0127 wt% when the mass ratio was 0.2:1 (Figure 4). Therefore, we selected MIL-101(Fe)@Ag (Ag 0.0127 wt%) for further investigation.

#### 2.1.4. UV–vis Spectroscopy

**Figure 5:**
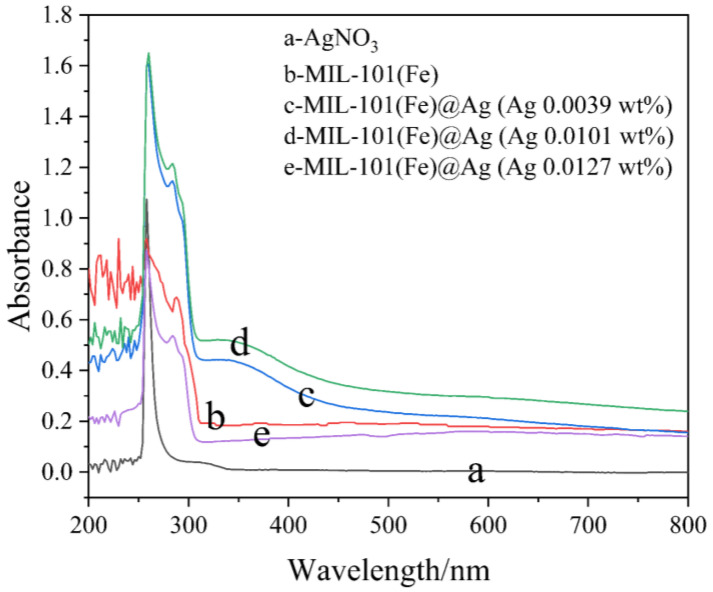
UV–vis spectra of AgNO_3_, MIL-101(Fe), and MIL-101(Fe)@Ag.

A Hitachi U-3010 UV–vis diffuse reflectance spectrophotometer was used to measure the UV–vis absorption spectra using DMSO as a reference [45]. We compared the UV–vis absorption spectra of MIL-101(Fe), MIL-101(Fe)@Ag, and silver nitrate, and we analyzed the preparation of MIL-101(Fe)@Ag with different silver nitrate contents using DMSO as a reference material. The UV–vis absorption spectra of MIL-101(Fe)@Ag, MIL-101(Fe), and silver nitrate are shown in Figure 5. The absorption peaks of MIL-101(Fe)@Ag were in the range of 260–290 nm, which was consistent with MIL-101(Fe). The absorption peak became higher and broader with the addition of Ag to MIL-101(Fe), covering the silver nitrate peak. MIL-101(Fe)@Ag showed significant absorption in the UV region (200–350 nm). In this experiment, the total amount of H_2_BDC and FeCl_3_·6H_2_O—the substrates used for synthesis of MIL-101(Fe)—was used as the standard, and the corresponding amount of silver nitrate was added to this total amount. The synthesized materials are called MIL-101(Fe)@Ag (Ag 0.0039 wt%), MIL-101(Fe)@Ag (Ag 0.0101 wt%), and MIL-101(Fe)@Ag (Ag 0.0127 wt%).

#### 2.1.5. ζ-Potential, Nanoparticle Size, and TG analysis of MIL-101(Fe)@Ag (Ag 0.0127 wt%)

**Figure 6:**
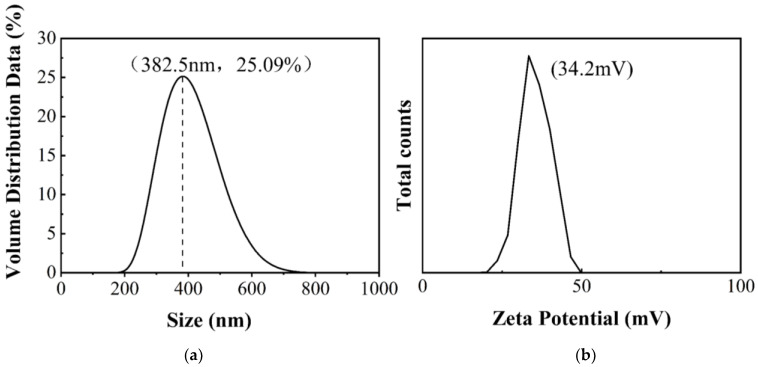
(**a**) Particle-size distribution and (**b**) zeta potential of MIL-101(Fe)@Ag (Ag 0.0127 wt%).

**Figure 7:**
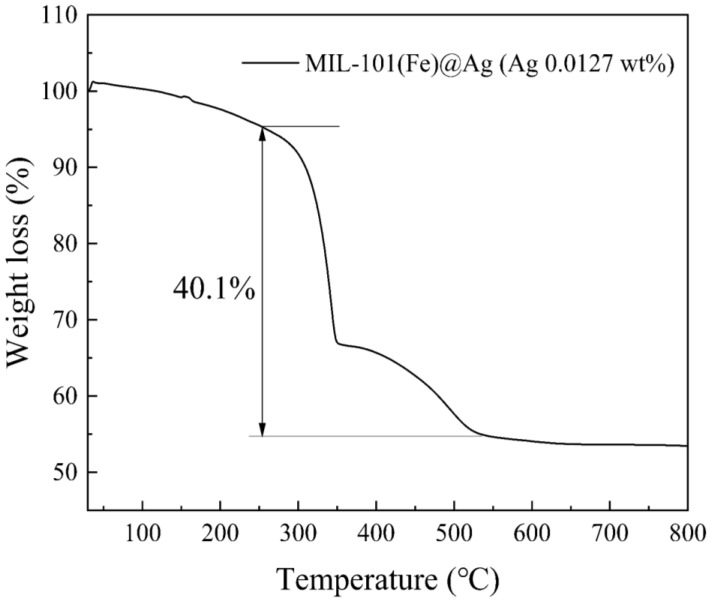
TG curve of MIL-101(Fe)@Ag (Ag 0.0127 wt%).

MIL-101(Fe)@Ag (Ag 0.0127 wt%) was further investigated. The ζ-potential of MIL-101(Fe)@Ag (Ag 0.0127 wt%) was 34.4 mV (Figure 6). The distribution of particles is more stable for a more stable dispersion system of particles according to ζ-potential and nanoparticle-size tests. It is generally considered that the cut-off line of the particle-dispersion stability in the aqueous phase is ±30 mV. If all of the particles have a zeta potential higher than +30 mV or lower than −30 mV, the dispersion system should be relatively stable. TG analysis can be used to determine the thermal stability of a substance by measuring the mass of the sample in the heated state as a function of temperature [42,48]. Here, the TG curve of the sample can be roughly divided into three phases (Figure 7). The first stage occurred from 30 to 250 °C, where the sample lost from approximately 1% to 10% of its weight due to the removal of the residual guest molecules from the crystal structure. The second stage was from 250 to 550 °C, where the sample lost the most weight (approximately 40% of its weight), indicating the collapse of the crystal structure. The highest heat-resistance temperature of MIL-101(Fe)@Ag (Ag 0.0127 wt%) was 350 °C. The third stage was from 550 to 650 °C, where the sample lost little weight and the curve was flat. In TG analysis, the synthesized material showed excellent stability, so it is suitable for application at high temperatures.

#### 2.1.6. XPS Analysis

**Figure 8:**
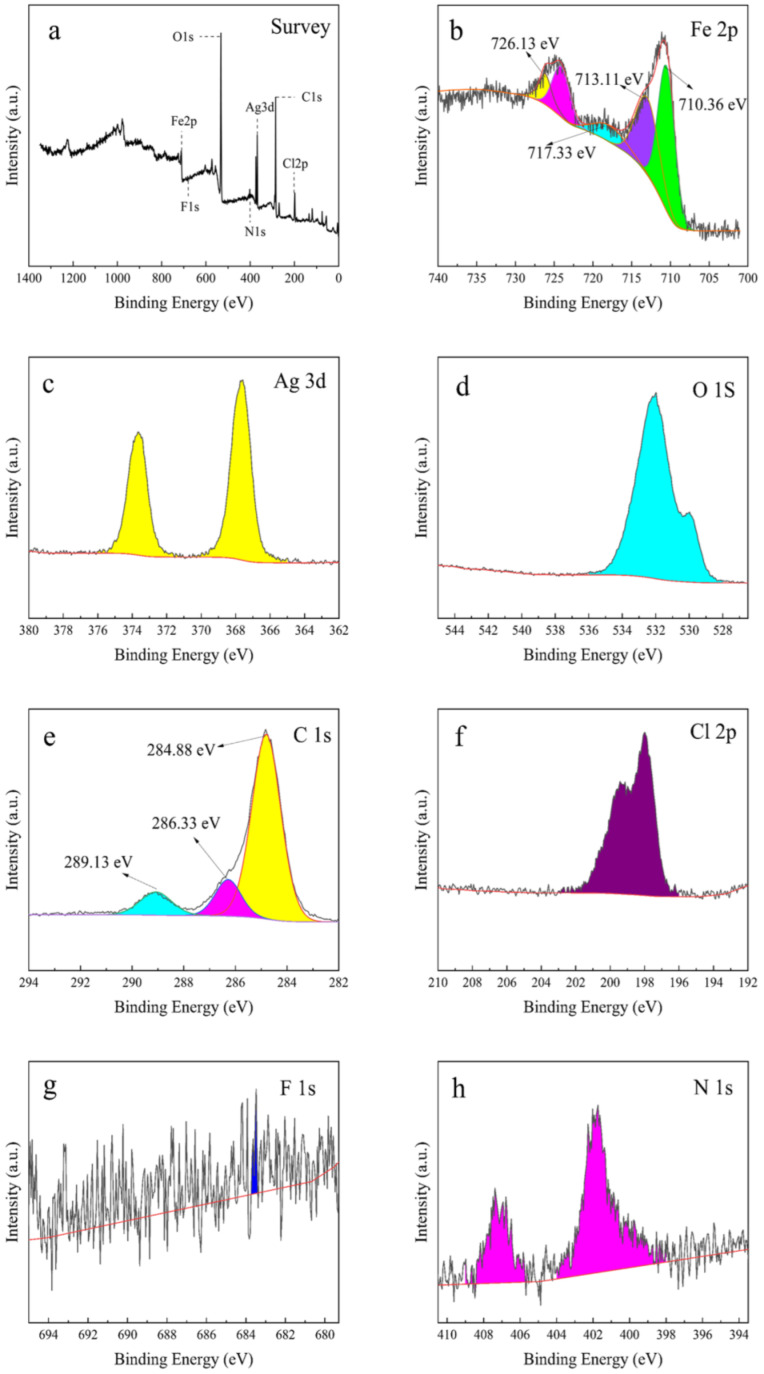
Elemental XPS spectra of MIL-101(Fe)@Ag (Ag 0.0127 wt%). (**a**) Full XPS spectrum of MIL-101(Fe)@Ag (Ag 0.0127 wt%). (**b**) XPS spectrum of Fe. (**c**) XPS spectrum of Ag. (**d**) XPS spectrum of O. (**e**) XPS spectrum of C. (**f**) XPS spectrum of Cl. (**g**) XPS spectrum of F. (**h**) XPS spectrum of N.

To further investigate the surface chemical composition and chemical valence of the complexes, the elements contained in the MIL-101(Fe)@Ag (Ag 0.0127 wt%) composite and the chemical composition were analyzed with XPS. XPS measurements provide information about elemental composition and chemical form, which can be obtained from the sample surface elemental content or concentration [43]. The results of XPS semi-quantitative analysis of MIL-101(Fe)@Ag (Ag 0.0127 wt%) are shown in Figure 8. The full spectrum of MIL-101(Fe)@Ag (Ag 0.0127 wt%) is shown in Figure 8a, indicating that MIL-101(Fe)@Ag (Ag 0.0127 wt%) was mainly composed of C, O, Fe, Cl, and Ag. The Fe 2p, C 1s, Ag 3d, and O 1s XPS spectra are shown in Figure 8b–f. The bond energies of Fe 2p were located at 710.36 and 726.13 eV (Figure 8b), which can be attributed to Fe 2p_3/2_ and Fe 2p_1/2_, respectively. Satellite peaks appeared at 713.11 and 717.33 eV, which are consistent with the trivalent Fe-binding form and indicate the successful construction of a bimetallic MOF structure containing +3 valent iron ions, which is consistent with previous results [44]. The high resolution C 1s spectrum was divided into three peaks (Figure 8c). The characteristic peaks at 284.088 and 289.13 eV can be attributed to the benzene ring and carboxylic acid groups on the organic ligand H_2_BDC in MIL-101(Fe), respectively [49].

#### 2.1.7. BET Analysis

**Figure 9:**
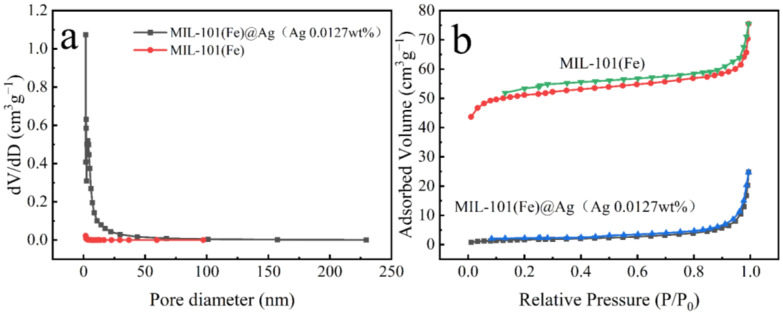
(**a**) Pore-size distributions of MIL-101(Fe)@Ag (Ag 0.0127 wt%) and MIL-101(Fe). (**b**) Adsorption and desorption curves of MIL-101(Fe)@Ag (Ag 0.0127 wt%) and MIL-101(Fe).

The BET surface area and pore structure were evaluated by performing N_2_ adsorption–desorption experiments. MIL-101(Fe) exhibited a typical type IV N_2_ adsorption–desorption curve (Figure 9), indicating that MIL-101(Fe) had a porous structure. The curve of MIL-101(Fe)@Ag (Ag 0.0127 wt%) was almost the same as that of MIL-101(Fe). The specific surface area and pore size analysis showed that the specific surface area of MIL-101(Fe) was 200 m^2^/g, the average pore diameter was 2.2 nm, and the pore volume was 0.1 cm^3^/g. After adding Ag^+^, the specific surface area of MIL-101(Fe)@Ag (Ag 0.0127 wt%) was 6 m^2^/g, the average pore diameter was 26 nm, and the pore volume was 0.04 cm^3^/g. The smaller specific surface area, smaller pore volume, and larger pore size of MIL-101(Fe)@Ag (Ag 0.0127 wt%) are believed to be due to the fact that even though the pore size in the synthesized material did not change, the pore size was blocked due to the addition of silver ions, which were embedded in the structure [50].

#### 2.1.8. SEM and EDS Analysis

**Figure 10:**
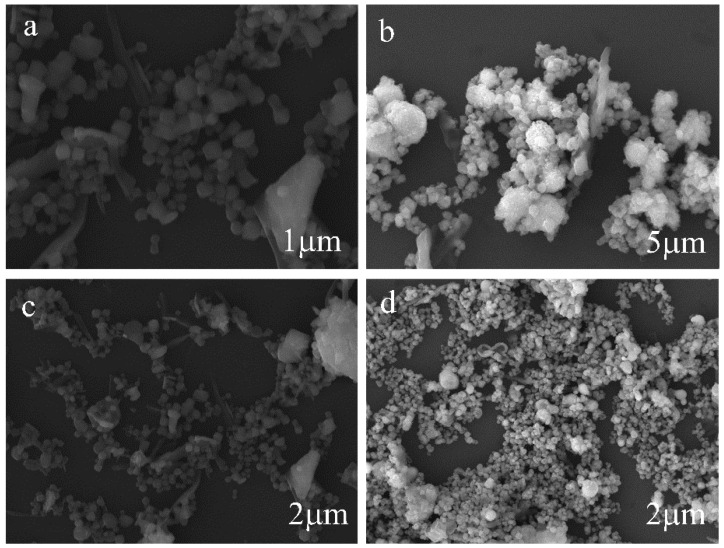
SEM images of (**a**,**c**) MIL-101(Fe) and (**b**,**d**) MIL-101(Fe)@Ag (Ag 0.0127 wt%).

**Figure 11:**
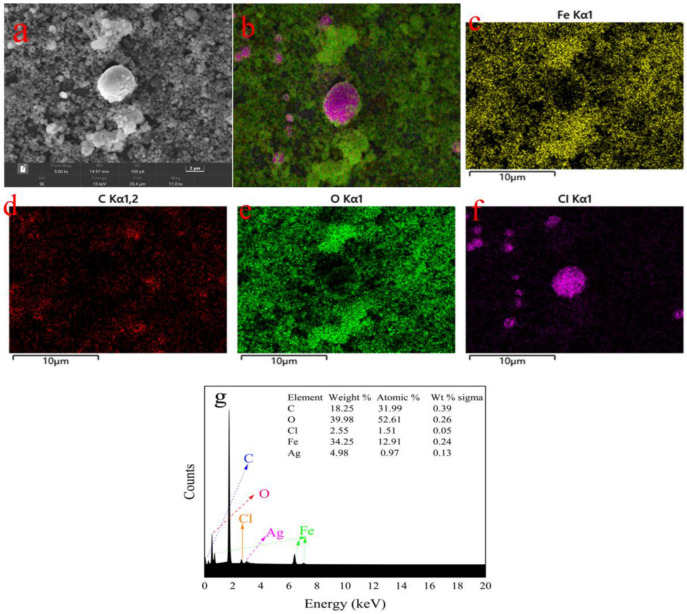
SEM images and EDS spectral analysis of MIL-101(Fe)@Ag (Ag 0.0127 wt%). Electron microscope images of MIL-101(Fe)@Ag (**a**–**f**) are energy dispersive X-ray spectroscopy (EDS) for elemental color mapping (Fe, C, O, Cl) spectrum; (**g**) is the energy absorption ratio of each element.

The morphology, shape, and size of the MIL-101(Fe) and MIL-101(Fe)@Ag samples were observed with SEM at an accelerating voltage of 200 eV to 30 keV (Figure 10). SEM has high resolution, and the depth of field is much greater than that of optical microscopy at the same magnification, thus enabling the observation of the three-dimensional structure. MIL-101(Fe) showed a six-hole stereoscopic structure with a uniform dispersion (Figure 10a,b). Combined with the abovementioned PXRD results, these results demonstrate that MIL-101(Fe) was successfully synthesized. An in-depth study was performed with EDS and elemental mapping (Figure 11). The elements C, O, F, Cl, and Fe were present in MIL-101(Fe)@Ag. The distributions of the elements C, O, and Fe showed the MIL-101(Fe) skeleton centered on trivalent iron, while the distributions of the elements F and Cl indicated that silver ions were embedded in the skeleton in the form of spheres. These results are consistent with the abovementioned XRD results and further confirm the synthesis of MIL-101(Fe)@Ag.

A component known to play an antibacterial role in similar structures is that of silver ions or nanosilver. Thus, in this study, the structure with the highest silver content, MIL-101(Fe)@Ag (Ag 0.0127 wt%), was used, and the results of experiments using MIL-101(Fe)@Ag (Ag 0.0127 wt%) and MIL-101(Fe) were compared.

### 2.2. Effect of MIL-101(Fe)@Ag (Ag 0.0127 wt%) on Bacterial Growth

#### 2.2.1. Inhibition-Zone Experiment

The concentration strongly affects the properties of nanomaterials. Thus, we analyzed the effect of different concentrations of MIL-101(Fe)@Ag (Ag 0.0127 wt%) on bacterial growth. We investigated the effect of MIL-101(Fe)@Ag (Ag 0.0127 wt%) particles on bacterial growth under normal conditions and with weak magnetic properties. According to inhibition-zone experiments of five different types of bacteria, MIL-101(Fe)@Ag (Ag 0.0127 wt%) had an inhibitory effect on all five bacteria and the presence of the inhibition zone clearly explained the inhibitory effect of MIL-101(Fe)@Ag (Ag 0.0127 wt%) on bacteria (Table 1).

#### 2.2.2. Determination of the Bacterial Growth Curves

**Figure 12:**
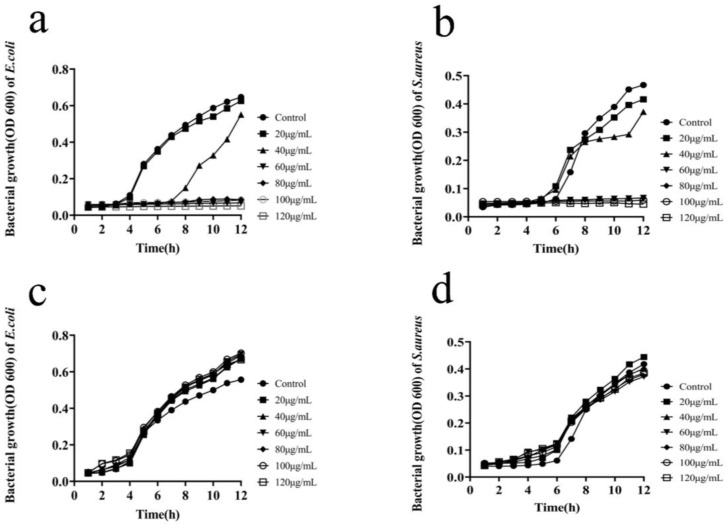
Bacterial growth curves of MIL-101(Fe) and MIL-101(Fe)@Ag (Ag 0.0127 wt%) for inhibition of *E. coli* and *S. aureus* growth. (**a**) Bacterial growth curve of inhibition of *E. coli* growth by MIL-101(Fe)@Ag (Ag 0.0127 wt%). (**b**) Bacterial growth curve of inhibition of *S. aureus* growth by MIL-101(Fe)@Ag (Ag 0.0127 wt%). (**c**) Bacterial growth curve of inhibition of *E. coli* growth by MIL-101(Fe). (**d**) Bacterial growth curve of inhibition of *S. aureus* growth by MIL-101(Fe).

The analysis of inhibition of bacterial growth by MIL-101(Fe)@Ag (Ag 0.0127 wt%) showed that bacterial inhibition became more pronounced as the silver content increased (Figure 12). *E. coli* and *S. aureus* are the two main bacteria commonly found in our lives, and they are Gram-negative and Gram-positive bacteria, respectively. *E. coli* is a normal host bacterium in the intestinal tract of animals, and a small percentage of *E. coli* causes diseases under certain conditions. Some serotypes of *E. coli* can cause human or animal gastrointestinal tract infections, which are mainly caused by specific bacterial hair antigens, pathogenic toxins, and other infections, as well as urinary tract infections, arthritis, meningitis and septicemia-type infections. *S. aureus* belongs to the genus Staphylococcus, and it is a common foodborne pathogenic microorganism. *S. aureus* is often parasitic in human and animal skin, the nasal cavity, the throat, and the gastrointestinal tract, and it is also ubiquitous in carbuncles, septic sores in the mouth, the air, sewage and other environments. Therefore, it is meaningful to use synthetic bimetallic MOF materials to inhibit bacterial growth. The growth curves of *E. coli* and *S. aureus* under normal conditions clearly show four periods of bacterial growth: delayed, logarithmic, stable, and decaying periods [51,52,53,54,55]. The progressive decrease in the logarithmic phase for different concentrations of MIL-101(Fe)@Ag (Ag 0.0127 wt%) (60, 80, 100 and 120 μg/mL), along with the decrease in the time for the bacteria to reach the plateau phase (Figure 12a,b), indicated that the inhibition of *E. coli* and *S. aureus* by MIL-101(Fe)@Ag (Ag 0.0127 wt%) was concentration-dependent. The OD600 values of untreated *E. coli* and *S. aureus* reached the logarithmic phase after 6 h. The OD600 value of the latter was found to be lower than that of the former for bacteria treated with MIL-101(Fe)@Ag (Ag 0.0127 wt%). The inhibition of bacteria became more pronounced as the concentration of MIL-101(Fe)@Ag (Ag 0.0127 wt%) increased [56,57,58]. This indicates that as the concentration of MIL-101(Fe)@Ag (Ag 0.0127 wt%) increased, the bacteria became less viable and the antibacterial effect of the material became stronger. The bacterial growth curves of MIL-101(Fe) showed that MIL-101(Fe) did not inhibit bacterial growth at concentrations of 0, 20, 40, 60, 80, 100 and 120 μg/mL (Figure 12c,d).

#### 2.2.3. Determination of the Bacterial Survival Rate

**Figure 13:**
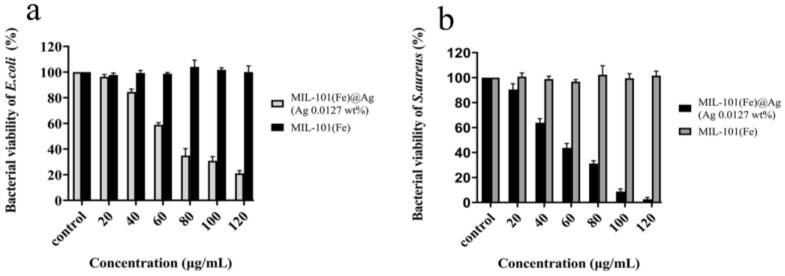
Results of plate-coating experiments with addition of MIL-101(Fe)@Ag (Ag 0.0127 wt%) and MIL-101(Fe) for inhibition of (**a**) *E. coli*. and (**b**) *S. aureus*.

The antibacterial performance of the synthesized products was tested with the plate-coating method (Figure 13a,b). The inhibition effect became more obvious with increasing concentrations of MIL-101(Fe)@Ag (Ag 0.0127 wt%), and the inhibition effect was proportional to the concentration of MIL-101(Fe)@Ag (Ag 0.0127 wt%). At a concentration of 100 μg/mL, MIL-101(Fe)@Ag demonstrated the significant inhibition of *E. coli*, and the survival rate of *S. aureus* was also lower than 10%. The experimental results showed that MIL-101(Fe)@Ag (Ag 0.0127 wt%) had an antibacterial effect on *E. coli* and *S. aureus*.

From the bacterial growth curves and plate-coating tests, we found differences in the antibacterial efficacy of MIL-101(Fe)@Ag (Ag 0.0127 wt%) against *S. aureus* and *E. coli*, namely, it was more effective for *S. aureus* than for *E. coli*. We speculate that this may have been because of the presence of pods in *E. coli*, which slowed the entry of MIL-101(Fe)@Ag (Ag 0.0127 wt%) into the bacteria and did not disrupt the cell membrane inside the bacteria, so the inhibitory effect was lower than that of *S. aureus*.

### 2.3. Antibacterial Mechanism of MIL-101(Fe)@Ag (Ag 0.0127 wt% Ag)

**Figure 14:**
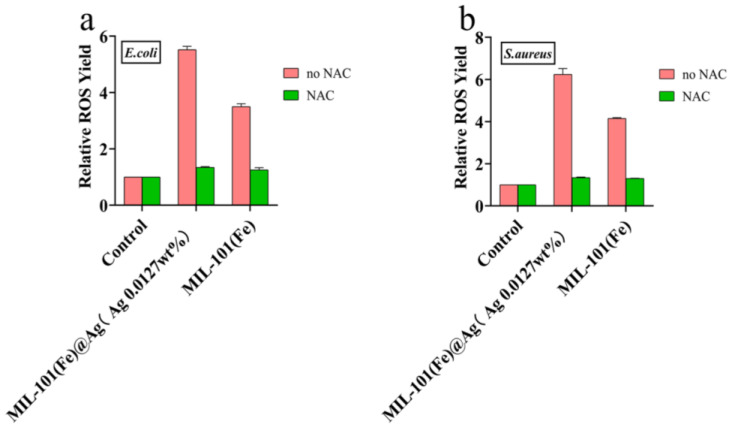
Intracellular ROS production by *E. coli* (**a**) and *S. aureus* (**b**) induced by treatment with MIL-101(Fe) and MIL-101(Fe)@Ag (Ag 0.0127 wt%). The content of ROS in all of the treatment groups was normalized to 1 with the control group.

To investigate how MIL-101(Fe)@Ag (Ag 0.0127 wt%) inhibits bacterial growth, the mechanism of inhibition was investigated. It is generally believed that bacterial growth is inhibited and bacteria are killed due to rupture of the bacterial cell membranes, thus leading to endoleaks, which results in the production of ROS and various free radicals [21,46]. ROS production has been found for various metal compounds, and these nanoparticles can lead to oxidative stress, inflammation, and consequent damage to proteins, cell membranes, and DNA, which is one of the main mechanisms of nanotoxicity [47]. The results of plate-coating experiments (Figure 14) revealed that bacteria treated with MIL-101(Fe)@Ag (Ag 0.0127 wt%) produced more ROS than those treated with MIL-101(Fe), and few ROS were produced by bacteria not treated with MIL-101(Fe)@Ag (Ag 0.0127 wt%), indicating that the bacterial cell membranes treated with MIL-101(Fe)@Ag were disrupted and produced more ROS [32,59,60].

### 2.4. Cytotoxicity Test

To provide the corresponding theoretical support for the further development and application of MIL-101(Fe)@Ag (Ag 0.0127 wt%), we investigated the biosafety of MIL-101(Fe) and MIL-101(Fe)@Ag (Ag 0.0127 wt%) with cytotoxicity and hemolysis experiments, which are described in Section 3.6 and Section 3.7, respectively [61,62,63,64].

Nanosilver has a damaging effect on cells above a certain concentration, and it affects the cell morphology and cell activity of erythrocytes. The addition of a small amount of silver ions to cells (200 μg/mL, higher than the minimum concentration for bacterial inhibition) resulted in an excellent antimicrobial effect. In addition, the cells treated with MIL-101(Fe)@Ag (Ag 0.0127 wt%) in this concentration range still had higher than 70% cell viability after 24 h, and the cell viabilities at 50 and 100 μg/mL were higher than those for MIL-101(Fe) without the addition of silver ions. In experiments with chicken erythrocytes, MIL-101(Fe)@Ag (Ag 0.0127 wt%) did not cause serious hemolytic effects, indicating that MIL-101(Fe)@Ag (Ag 0.0127 wt%) is non-toxic or hypotoxic to cells at less than 200 μg/mL [65,66].

**Figure 15:**
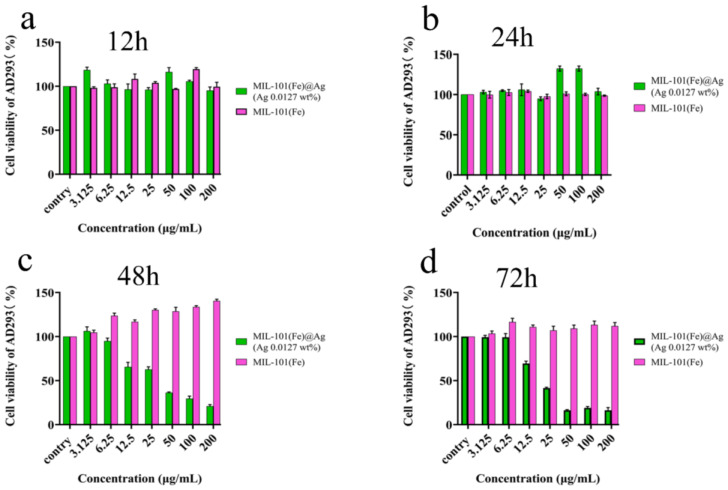
Survival rate of MIL-101(Fe)@Ag -treated AD293 cells after 12 h (**a**), 24 h (**b**), 48 h (**c**), and 72 h (**d**).

Cellular compatibility is a crucial issue for Ag-containing materials [67]. When AD293 cells were treated with different concentrations of MIL-101(Fe)@Ag (Ag 0.0127 wt%) pellets, the results of MTT experiments showed no significant differences in cell survival, and cell survival was maintained at more than 90% (Figure 15). Thus, the AD293 cell-viability experiments under the influence of MIL-101(Fe)@Ag (Ag 0.0127 wt%) revealed the non-toxic nature of MIL-101(Fe)@Ag (Ag 0.0127 wt%). Because MIL-101(Fe)@Ag (Ag 0.0127 wt%) is non-toxic or hypotoxic to cells, it can be used in biological applications and will further extend the use of synthetic bimetallic MOFs [68].

**Figure 16:**
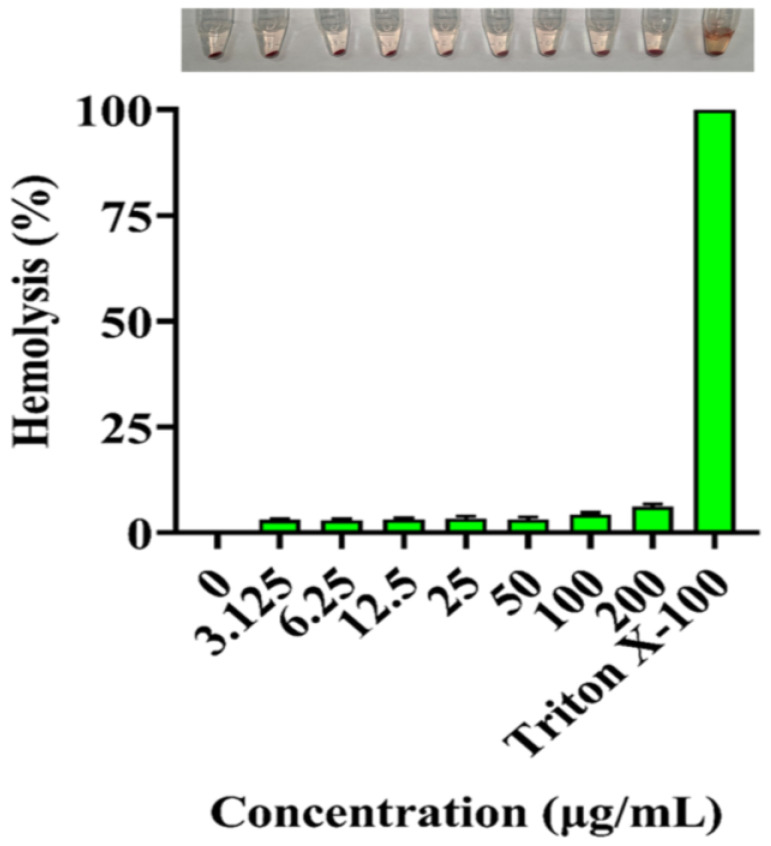
Hemolysis analysis of MIL-101(Fe)@Ag (Ag 0.0127 wt%). Physiological saline and 0.1% Triton X-100 were used as negative and positive controls, respectively.

The hemolytic behavior of MIL-101(Fe)@Ag (Ag 0.0127 wt%) was also investigated to assess its biocompatibility. In general, hemoglobin is released via the disruption of the red-blood-cell membrane, thus allowing hemolysis to occur. MIL-101(Fe)@Ag (Ag 0.0127 wt%) did not produce severe hemolytic effects on chicken erythrocytes, even as the concentration of MIL-101(Fe)@Ag (Ag 0.0127 wt%) increased (Figure 16). Similar to the results of the cytotoxicity assay, the results of hemolysis analysis showed that high doses of MIL-101(Fe)@Ag (Ag 0.0127 wt%) did not cause severe hemolytic effects [61,62].

## 3. Experimental

### 3.1. Materials

Dulbecco’s modified Eagle medium, phosphate-buffered pH 7.4 (1×) 0.25% trypsin-EDTA (1×), and penicillin–streptomycin (100,000 U/mL) were purchased from Gibco (Carlsbad, CA, USA). Fetal bovine serum was obtained from Shanghai Exel Biologicals (Shanghai, China). Silver nitrate (AgNO_3_, 98%) was obtained from Shanghai Malin Biologicals (Shanghai, China). Terephthalic acid (H_2_BDC), ferric chloride hexahydrate (FeCl_3_·6H_2_O), *N*,*N*-dimethylformamide (DMF), and chicken erythrocytes were purchased from GBCBIO Technologies (Guangzhou, China). The culture flasks were obtained from Corning Life Sciences Ltd. (New York, NY, USA). The 96-well plates were purchased from Guangzhou JITE Biofiltration Co. (Guangzhou, China). Glycerol (99%) was purchased from Aladdin Reagents Ltd. (Shanghai, China). Luria–Bertani (LB) broth and nutritional agar medium were purchased from Guangdong Huan Kai Microbial Technology Co. (Guangzhou, China). The hydrothermal synthesis reactor was purchased from Zhengzhou Boke Instrument & Equipment Co. (Zhengzhou, China). The electrothermal blast thermostat oven (101-0B) was purchased from Shaoxing Licheng Instrument Technology Co. (Shaoxing, China). The Thermo-SorvallST16R frozen centrifuge and ABI7500 real-time quantitative PCR instrument were purchased from Thermo Fisher Scientific (Waltham, MA, USA).

### 3.2. Preparation of MIL-101(Fe) and MIL-101(Fe)@Ag

Based on the synthesis method of MIL-101(Fe) reported in the literature [31], the synthesis of MIL-101(Fe) and MIL-101(Fe)@Ag was further optimized using the following method: 1.351 g of FeCl_3_∙6H_2_O (5 mmol) and 0.415 g (2.5 mmol) of H_2_BDC were added to 30 mL of DMF. The solution was then sonicated for 15 min to make it cloudy, followed by the addition of a certain mass of silver nitrate (H_2_BDC + FeCl_3_·6H_2_O mass percentages of 0%, 5%, 10%, and 20%) as anhydrous ethanol containing silver nitrate to 6 mL of the suspension. The suspension was sealed in a stainless-steel reactor lined with polytetrafluoroethylene and heated at 120 °C for 24 h to obtain an orange slurry. The orange slurry was centrifugally separated (4000 rpm for 10 min), washed once with DMF and twice with hot ethanol at 60 °C, and centrifuged (4000 rpm for 15 min) to remove unreacted raw materials. The washed orange slurry was kept at 150 °C for 12 h to activate the slurry. The finally obtained MIL-101(Fe)@Ag was an orange powder.

### 3.3. Nanomaterial Characterization

Powder XRD (Rigaku Ultima IV, Kyoto, Japan) was performed using Cu-Kα radiation in the 2θ range from 5° to 80°. For FTIR spectroscopy (Bruker ALPHA II, Karlsruhe, Germany), the spectra of MIL-101(Fe) and MIL-101(Fe)@Ag were recorded in the wavelength range of 500–4000 cm^−1^. An inductively coupled plasma (ICP) spectrometry generator was used to determine the Ag content in MIL-101(Fe)@Ag. The ultraviolet–visible (UV–vis) absorption spectra were measured with a HITACHI U-3010 UV–vis diffuse reflectance spectrophotometer (Hitachi Limited, Kyoto, Japan) using dimethyl sulfoxide (DMSO) as a reference. The thermogravimetric (TG) analysis of MIL-101(Fe)@Ag (NETZSCH STA 2500, Germany) was performed at a target temperature 800 °C with a heating rate of 10 °C min^−1^ and an airflow rate of 100 mL min^−1^ in an air atmosphere. The zeta potential and particle-size distribution were measured with a Malvern Zetasizer Nano ZS90 instrument (Malvern, UK). X-ray photoelectron spectroscopy (XPS, K-Alpha+, Thermo Fisher Scientific) was performed to study the chemical composition, chemical states, and valence band of MIL-101(Fe)@Ag using Al Kα radiation. The morphology, shape, and size of MIL-101(Fe) and MIL-101(Fe)@Ag were investigated with scanning electron microscopy (SEM, TESCAN Mira4, Czech Republic) at an accelerating voltage of 200 eV to 30 keV. The chemical composition of MIL-101(Fe)@Ag was further analyzed with energy dispersive X-ray spectrometry (EDS, Zeiss EVO, Oberkochen, Germany). The Brunauer–Emmett–Teller (BET) surface area and porous structure were evaluated with N_2_ (77.4 K) adsorption–desorption experiments (Micromeritics ASAP 2020 v4.03, Norcross, GA, USA).

### 3.4. Antimicrobial Activity

#### 3.4.1. Inhibition-Zone Experiment

A circular piece of paper with a diameter of 9 mm was dried by heating in a autoclave and set aside. The prepared MIL-101(Fe)@Ag was dispersed in ultrapure water, soaked for 1 h, removed, and dried. The bacterial solution (5 × 10^7–8^ CFU/mL, 100 μL) was spread evenly on the plate. The dried drug-sensitive paper was spread on the plate, and the antimicrobial ring formed was observed after incubation in a 37 °C light incubator for 24 h [32].

#### 3.4.2. Determination of the Bacterial Growth Curve

An *E. coli* and *S. aureus* stock solution (10 μL) was dispersed in 10 mL of LB broth, observed, and then incubated for 12 h in a thermostatic shaker at 37 °C. The bacteria were then diluted to 1 × 10^6^ CFU/mL with LB broth.

Different concentrations of MIL-101(Fe)@Ag (0, 20, 40, 60, 80, 100, and 120 μg/mL) were added to 100 μL of the diluted bacteria and cultured at 37 °C. Samples were taken at different times (1–12 h), and the optical density at 600 nm (OD_600_) value of the bacteria was calculated with a microplate reader [33,55].

#### 3.4.3. Determination of the Bacterial Survival Rate

Bacteria (100 μL, 1 × 10^6^ CFU/mL) with different concentrations of MIL-101(Fe)@Ag were incubated at 37 °C for 3 h. Next, 10 μL of the diluted bacterial solution was applied to an agar plate. The agar plate was then incubated overnight in a constant temperature incubator. Finally, the number of colonies on the agar plate was counted, and the antibacterial effect of MIL-101(Fe)@Ag was evaluated with the following equations:

Survival rate (%) = CFU (experimental group)/CFU (control group) × 100%(1)

Mortality rate (%) = 1 − survival rate (%)(2)

where CFU (experimental group) is the number of colonies in the material treatment group and CFU (control group) is the number of colonies in the control group [34,35].

### 3.5. Investigation of the Antibacterial Mechanism

The determination of the ROS in the bacterial cells was performed with an ROS kit from the Nanjing Jiancheng Institute of Biological Engineering [32]. First, a certain concentration of bacteria (1.0 × 10^6^ CFU/mL) was mixed with 20% MIL-101(Fe)@Ag/MIL-101(Fe) and incubated at 37 °C for 2 h. The bacteria were collected via centrifugation, washed three times with phosphate-buffered saline, treated with 2′,7′-dichlorofluorescein diacetate, and incubated for 1 h at room temperature protected from light. After incubation, the bacteria were centrifuged at 3000 rpm for 5 min, phosphate-buffered saline was added to dilute the bacteria to the initial volume, and the bacterial solution was resuspended. A Shimadzu RF-5301 PC fluorescence spectrophotometer was then used to measure the fluorescence intensity of the bacterial suspension. The excitation and emission wavelengths were 488 and 525 nm, respectively [36,37].

### 3.6. Cytotoxicity Test

The cytotoxicity of MIL-101(Fe)@Ag was determined with an MTT assay [33]. AD293 cells were digested by trypsin and inoculated in 96-well cell culture plates at a density of 1 × 10^5^/well, and then the well plates were incubated in a cell-culture incubator (37 °C and 5% CO_2_) for culturing. After the cells at the bottom of the well plate grew all over the bottom of the culture flask, the culture medium in the wells was removed and 100 μL of different concentrations of MIL-101(Fe)@Ag was added to each well. Four replicate wells for each MIL-101(Fe)@Ag concentration, as well as a blank control group, were set up, and then culturing was continued in the incubator for 24 h to observe the cytopathological changes.

After 24 h of incubation, 20 μL of MTT solution (5 mg/mL) was added to the wells and incubation was continued for 4 h. After incubation, the supernatant was carefully removed and 150 μL of DMSO was added to each well to dissolve the bottom methanogens. The well plate was then placed on a shaker to shake the well solution, and the absorbance at 492 nm was finally measured by an enzyme marker. The cell-survival rate was calculated with the following equation:

Cell viability (%) = OD492 of the sample wells/OD492 of the blank wells × 100%(3)

### 3.7. Hemolytic Assay

To further evaluate the biocompatibility of MIL-101(Fe)@Ag, we determined its hemolysis rate. Chicken erythrocytes were collected via centrifugation at 2000 rpm, washed three times with saline (0.9% NaCl), and resuspended in saline to make a 2% suspension of chicken erythrocytes. Different concentrations of MIL-101(Fe)@Ag were mixed with equal volume of the erythrocyte suspension so that the final concentrations of MIL-101(Fe)@Ag were 0, 3.125, 6.25, 12.5, 25, 50, 100, and 200 μg/mL, followed by incubation at 37 °C for 2 h. Physiological saline and 1% tretinoin X-100 were used as negative and positive controls, respectively, in simultaneous experiments. After incubation, the supernatant was centrifuged at 3000 rpm for 5 min. The supernatant was then transferred to a clean 96-well plate with four replicate wells for each sample, and the UV absorbance intensity at 450 nm was measured with an enzyme marker [38,39]. The hemolysis rate was calculated as:

Hemolysis rate (%) = (absorbance of the experimental group − absorbance of the negative control group)/(absorbance of the positive control group − absorbance of the negative control group) × 100%.

### 3.8. Statistical Analysis

All of the experiments were performed in triplicate. The data are expressed as the mean ± standard deviation. Statistical significance was assessed using the Student’s *t*-test, and the values were considered to be significant at *p* < 0.05.

## 4. Conclusions

In this study, we synthesized the target product MIL-101(Fe)@Ag with solvothermal synthesis. We evaluated the antibacterial activity and biosafety of MIL-101(Fe)@Ag (Ag 0.0127 wt%). Characterization by FTIR spectroscopy, SEM, particle-size and zeta-potential analysis, PXRD, and TG analysis revealed that MIL-101(Fe) was successfully loaded with Ag^+^ and that 0.0127 wt% silver nitrate was the highest amount that could be loaded in MIL-101(Fe)@Ag without silver-ion aggregation and structure collapse [34,35,37,38,40]. Because the antibacterial effect of MIL-101(Fe)@Ag (Ag 0.0127 wt%) and its mechanism occur through the interaction of the silver ions in MIL-101(Fe)@Ag (Ag 0.0127 wt%) with oxygen in water or air under light conditions, the ROS O^2−^ and HO∙ are produced. These ions have an extremely high redox effect and can destroy the cell membrane of bacteria in a short period of time, rendering the bacteria inactive. Accordingly, the aim of inhibiting bacterial growth was achieved. The silver ions in MIL-101(Fe)@Ag (Ag 0.0127 wt%) act as a catalyst and are not consumed, so MIL-101(Fe)@Ag (Ag 0.0127 wt%) has a long-lasting antibacterial effect. In cytotoxicity tests, as the concentration of MIL-101(Fe)@Ag (Ag 0.0127 wt%) gradually increased, the survival rate of AD293 cells also gradually decreased but the cell viability remained high. The cell-survival rate remained above 75%, indicating that MIL-101(Fe)@Ag (Ag 0.0127 wt%) is non-toxic to AD293 cells. These phenomena also indicate that MIL-101(Fe) and MIL-101(Fe)@Ag (Ag 0.0127 wt%) have good biocompatibility [48,49,50,51,61,62,68]. However, the bound MIL-101(Fe)@Ag is still limited in concentration due to the inherent toxicity of silver ions [18]. As a common biomolecular nanosystem, such as antimicrobial peptides, its biosafety is better than MIL-101(Fe)@Ag [26,27]. However, the antibacterial effect of nanomolecular systems composed of chemical materials is better than that of common biological nanosystems, and its structure is extremely stable, which is conducive to efficient antibacterial [52,61]. Other drug-loaded bio-nano-molecular systems have high safety for organisms, but their main antibacterial effects are slightly worse than our synthesized MIL-101(Fe)@Ag. For example, the MIL-101(Fe)-T705 synthesized on the basis of MIL-101(Fe) has a higher bacteriostatic MIC value (3.2 mg/mL) against *Staphylococcus aureus* than MIL-101(Fe)@Ag (100 µg/µL) [68].

MIL-101(Fe)@Ag (Ag 0.0127 wt%) showed excellent antimicrobial properties against both *S. aureus* and *E. coli*. In terms of the antibacterial mechanism, the presence of Ag^+^ disrupts the bacterial cell membrane, resulting in the bacterial DNA being unable to replicate and making bacterial proliferation impossible. The results indicated that MIL-101(Fe)@Ag (Ag 0.0127 wt%) is a promising antimicrobial material for biomedical use, and coupled with its inherent nontoxicity, it is expected to be further applied in tissue-engineering materials, drug carriers, antimicrobial products, and other similar fields.

## Figures and Tables

**Table 1:** Inhibition-zone diameters of MIL-101(Fe) and MIL-101(Fe)@Ag (Ag 0.0127 wt%) (mm).

Types of Bacteria	MIL-101(Fe)@Ag (Ag 0.0127 wt%)	MIL-101(Fe)
*Escherichia coli*	12	9
*Staphylococcus aureus*	12.3	9
*Staphylococcus epidermidis*	10	9
*Acinetobacter cereus*	11	9
*Acinetobacter jungii*	11	9
*Pseudomonas aeruginosa*	11	9

## Data Availability

The data presented in this study are available on request from the corresponding author.
